# Deciphering the mechanisms of long non-coding RNAs in ferroptosis: insights into its clinical significance in cancer progression and immunology

**DOI:** 10.1038/s41420-025-02290-6

**Published:** 2025-01-18

**Authors:** Shengming Ou, Xiaoya Nie, Xiangyu Qiu, Xin Jin, Geyan Wu, Rongxin Zhang, Jinrong Zhu

**Affiliations:** 1 Guangdong Provincial Key Laboratory of Advanced Drug Delivery, Guangdong Provincial Engineering Center of Topical Precise Drug Delivery System, School of Life Sciences and Biopharmaceutics, Guangdong Pharmaceutical University, Guangzhou, China; 2 Biomedicine Research Centre, Guangdong Provincial Key Laboratory of Major Obstetric Diseases; Guangdong Provicial Clinical Research Center for Obsterics and Gynecology, The Third Affiliated Hospital of Guangzhou Medical University, Guangzhou Medical University, Guangzhou, China

**Keywords:** Cell death, Cancer therapy

## Abstract

A new type of nonapoptotic, iron-dependent cell death induced by lipid peroxidation is known as ferroptosis. Numerous pathological processes, including inflammation and cancer, have been demonstrated to be influenced by changes in the ferroptosis-regulating network. Long non-coding RNAs (LncRNAs) are a group of functional RNA molecules that are not translated into proteins, which can regulate gene expression in various manners. An increasing number of studies have shown that lncRNAs can interfere with the progression of ferroptosis by modulating ferroptosis-related genes directly or indirectly. Despite evidence implicating lncRNAs in cancer and inflammation, studies on their mechanisms and therapeutic potential remain scarce. We investigate the mechanisms of lncRNA-mediated regulation of inflammation and cancer immunity, assessing the feasibility and challenges of lncRNAs as therapeutic targets in these conditions.

## Facts

- Ferroptosis is associated with the development of a variety of diseases, such as inflammation and cancer.
- Inducing ferroptosis holds the potential to treat drug-resistant tumors and enhance the efficacy of immunotherapy.
- The aberrant expression of lncRNAs is widely acknowledged for its profound impact on oncogenic processes.
- Ferroptosis and lncRNA can regulate the secretion of cytokines, thereby affecting the progression of diseases such as inflammation and cancer.
- LncRNA expression is tissue-specific and stage-specific and can be used as a biomarker for disease diagnosis.

## Open questions

- What is the precise execution mechanism of ferroptosis? Are there any unknown mechanisms of ferroptosis induction?
- Ferroptosis is associated with cancer immunotherapy, but what is the specific mechanism of their interaction?
- How can the regulation of ferroptosis by lncRNA be harnessed to intervene in the course of diseases, thereby achieving the goal of treating diseases?
- How can the synergistic effect of lncRNA and ferroptosis be enhanced to improve the efficacy of cancer immunotherapy?
- How can specific lncRNAs be delivered via exosomes to regulate ferroptosis, and how can the efficacy and safety of this combined application be assessed?

## Introduction

In 2012, Dixon and his colleagues first proposed the concept of “ferroptosis”, indicating ferroptosis as a novel regulatory form of cell death dependent on iron-induced lipid peroxidation accumulation [1]. Compared to other types of regulated cell death such as apoptosis, autophagy, and necrosis, ferroptosis exhibits significant differences in morphology and mechanism. Its morphological features mainly include mitochondrial shrinkage, decreased mitochondrial cristae, and increased membrane density [1–3]. Biochemically, the key features of ferroptosis include increased reactive oxygen species (ROS) generation and accumulation of lipid peroxides. Ferroptosis plays a crucial role in various pathological processes such as inflammatory diseases [4]. Research on this form of cell death not only deepens our understanding of cellular fate regulation but also provides new directions for therapeutic strategies. Particularly noteworthy is the relationship between ferroptosis and tumor immunotherapy. Studies suggest that targeting ferroptosis may overcome resistance to certain cancer treatments, offering patients who are not responding well to conventional therapy with alternative therapeutic options [5–7].

Long non-coding RNAs (lncRNAs), exceeding 200 nucleotides in length, are a diverse class of transcripts encoded by the genome. An increasing body of research indicates that lncRNAs are capable of intricately regulating gene expression by engaging in complex interactions with DNA, mRNA, and proteins. Through these interactions, lncRNAs exert regulatory control over various aspects of cellular processes, including chromatin organization, transcriptional modulation, protein translation, and subcellular localization [8, 9]. Furthermore, lncRNAs serve as competitive endogenous RNAs (ceRNAs) for microRNAs (miRNAs), thereby modulating gene expression levels indirectly through competitive binding with target gene mRNAs. This mechanism introduces an additional stratum of intricacy within the regulatory framework, thereby elucidating the polyvalent functions of lncRNAs in the modulation of gene expression [10, 11]. Notably, a subset of lncRNAs harbors small open reading frames (sORFs), encoding functional peptides with tissue specificity implicated in various biological processes, including cancer progression [12–14]. Despite their generally low expression levels under normal physiological conditions, certain lncRNAs exhibit tissue-specific or condition-dependent upregulation [15], rendering them promising candidates as biomarkers and therapeutic targets. For instance, lncRNA PCA3 has emerged as a promising biomarker for early prostate cancer detection [16], and the aberrant expression of lncRNA MALAT1 in cancer cells underlines its potential as a therapeutic target in cancer treatment [17, 18]. In addition, numerous studies suggest that lncRNAs play a pivotal role in regulating ferroptosis. Research has revealed that LINC00618, a lncRNA, accelerates ferroptosis in a manner dependent on apoptosis, thereby influencing the ferroptosis process in cancer [19]. Additionally, lncRNAs play a crucial role in regulating inflammatory diseases and the immune system [20].

Elucidating the molecular mechanisms of lncRNAs in immune regulation can provide new strategies for the early diagnosis and treatment of cancer and inflammatory diseases. Therefore, this paper reviews the molecular mechanisms of lncRNAs in the immune system and inflammation, focusing on the role of lncRNAs in regulating cancer development via the ferroptosis pathway. This sheds light on targeting ferroptosis and ferroptosis-related lncRNAs as a potential clinical approach for cancer therapy.

## Metabolic mechanisms of ferroptosis

The cellular mechanisms underlying ferroptosis primarily rely on the generation and detoxification of lipid peroxidation, which are pivotal biological processes. In the event of cells being unable to effectively neutralise excessive intracellular ROS through antioxidant mechanisms, an accumulation of oxidised lipids occurs, which ultimately results in ferroptosis [21]. In this antioxidant biological process, glutathione peroxidase 4 (GPX4) and glutathione (GSH) play a significant regulatory role in conjunction with one another. Furthermore, the ferroptosis suppressor protein 1 (FSP1)-Coenzyme QH2 (CoQH2) and Dihydroorotate dehydrogenase (DHODH)-CoQH2 systems also exert indispensable effects [22, 23]. Furthermore, the onset of ferroptosis is associated with multiple cellular metabolic processes, including iron and lipid metabolism. In conclusion, the regulation of ferroptosis is a complex process involving a multitude of cellular metabolic pathways.

## Ferroptosis and inflammation

**Fig. 1:**
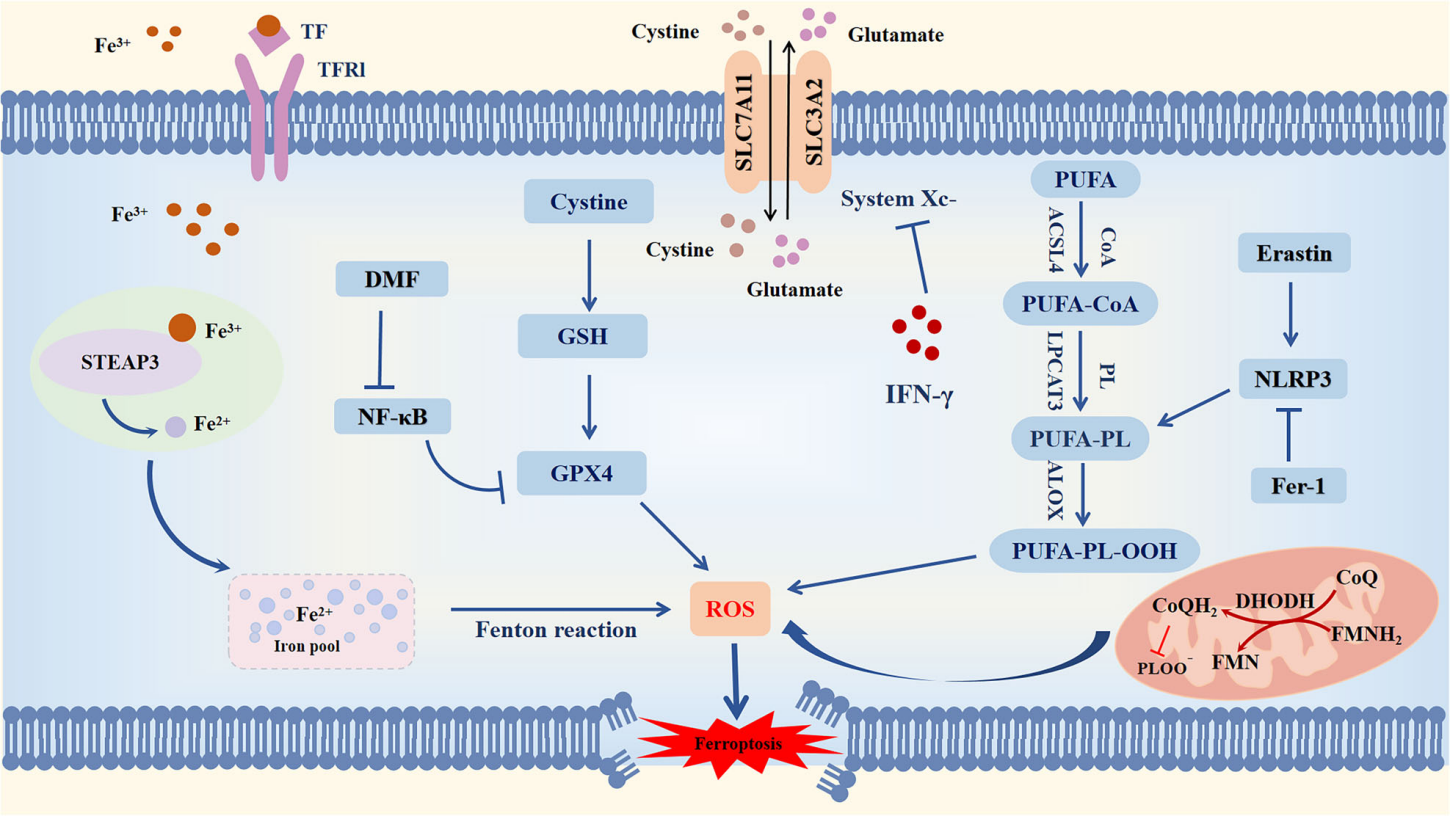
Interplay between inflammatory cytokines and ferroptosis. DHODH dihydroorotate dehydrogenase, FSP1 Ferroptosis suppressor protein 1, DMF Dimethyl fumarate, NF-κB nuclear factor kappa-light-chain-enhancer of activated B cells, GPX4 Glutathione peroxidase 4, ACSL4 Long-chain Acyl-CoA Synthetase 4, GSH Glutathione, LIP Labile iron pool, PUFA Polyunsaturated fatty acids, ROS Reactive oxygen species, TF Transferrin, TFR1 Transferrin receptor 1, STEAP3 Six-Transmembrane Epithelial Antigen of Prostate 3, NLRP3 Nucleotide-binding oligomerization structural domain receptor protein 3, FMN Flavin mononucleotide, LPCAT3 Lysophosphatidylcholine acyltransferase 3, ALOX Arachidonate 5-lipoxygenase.

Inflammation is a protective response to stimuli that disturb the homeostasis within the body [24]. In normal circumstances, inflammation promptly resolves after the body clears an infection, thereby restraining continued tissue damage during the inflammatory phase [25]. The relationship between inflammation and ferroptosis is highly intricate. When cells undergo ferroptosis, lipid peroxides on the membrane may be identified as damage-associated molecular patterns (DAMPs), thus instigating immune system activation and triggering an inflammatory response [26]. Oxidative stress and immune responses occurring during the inflammatory process may also contribute to the promotion of ferroptosis [27]. Several inflammatory cytokines [28], such as Interleukin-1 (IL-1), IL-6, and tumor necrosis factor (TNF) have been demonstrated to impact the levels and activity of GPX4. For instance, TNF treatment of cells can lead to decreased GPX4 levels, potentially further inducing ferroptosis. The nuclear factor kappa-light-chain-enhancer of activated B cells (NF-κB) is a canonical signaling pathway involved in various pathological and physiological processes [29], regulating inflammation and the immune system. Dimethyl fumarate (DMF), as an activator of nuclear factor erythroid 2-related factor 2(NRF2), leads to upregulation of IκBα and inhibition of NF-κB signaling pathway activation, promoting the expression of heme oxygenase 1(HMOX1) and GPX4. Consequently, this alleviates neuroinflammation and inhibits ferroptosis [30]. Nucleotide-binding oligomerization structural domain receptor protein 3 (NLRP3) inflammasome has been demonstrated to be associated with ferroptosis. In dry AMD mouse models, intracellular accumulation of excess Fe^2+^ drives NLRP3 inflammasome activation through the cGAS-STING1 pathway, inducing lipid peroxidation and consequently leading to ferroptosis [31]. Furthermore, ferroptosis inhibitor ferritin-1 suppresses the expression of NLRP3 and caspase-1, while ferroptosis inducer Erastin promotes the activation of NLRP1 and NLRP3 [32]. These studies suggest that inflammasomes may be pivotal steps in ferroptosis. Other inflammation-related signaling pathways, including MAPK, JAK/STAT, and cGAS-STING1, have also been demonstrated to be closely associated with ferroptosis [33]. Overall, the interplay between ferroptosis and these inflammatory signaling pathways provides novel strategies for the treatment of inflammatory diseases (Fig. 1).

CD8^+^ T cells and CD4^+^ T cells deficient in GPX4 exhibit impaired resistance against viral and parasitic infections [34]. HMGB1, a typical DAM, can engage pattern recognition receptors, thereby instigating the activation of innate and adaptive immunity cascades alongside eliciting inflammatory responses. The attenuation of HMGB1 release helps to reduce the inflammatory environment during the progression of ferroptosis [35]. Furthermore, HMGB1 has been intricately associated with diverse oncogenic advancements and unfavorable prognostic outcomes [36]. Consequently, meticulous scrutiny is warranted to validate the assertion positing HMGB1’s favorable influence on anti-tumor immunotherapeutic strategies propelled by ferroptosis.

## Ferroptosis and tumor immunity

Previous research has demonstrated that immune cells can trigger ferroptosis in tumor cells through the production of cytokines, thereby contributing to the development of an anti-tumor immune response. Ferroptosis also plays a role in the anti-tumor immune process [37]. According to Wang et al., the combination of ferroptosis inducers and immune checkpoint inhibitors can enhance T cell-mediated anti-tumor immune responses. This study further elucidates that anti-PD-L1 immunotherapy induces the release of TNFs and interferon-gamma (IFN-γ) by CD8^+^ T cells, thereby inhibiting the expression of Xc-system, promoting ferroptosis in tumor cells, and highlighting the pivotal role of ferroptosis in immune therapy [38, 39]. This revelation sheds light on a novel mechanism by which ferroptosis inhibits tumor cells. However, an alternative investigation posits that ferroptosis inducers possess the capability to induce apoptosis in T cells, consequently impeding immune responses. This indicates that the function of ferroptosis in regulating cancer immunotherapy is intricate and contingent upon the specific context. Moreover, it has been observed that ferroptosis exhibits synergistic effects between radiotherapy and immunotherapy [40]. Consequently, a deeper exploration into the disparate susceptibility of cancerous and immune cells to ferroptosis is imperative.

## The role of lncRNA in ferroptosis

LncRNAs have been demonstrated to play a role in the regulation of iron metabolism and other related genes through various mechanisms. Consequently, further investigation into the precise mechanisms by which lncRNAs regulate ferroptosis may facilitate the development of novel therapeutic strategies for disease treatment.

Recent studies have underscored the pivotal regulatory role of lncRNAs as facilitators of ferroptosis. For example, lncRNA RP11-89 has been implicated in the augmentation of PROM2-mediated formation of iron-laden multivesicular bodies (MVBs) and subsequent iron release by silencing miR-129-5p expression, culminating in fatal intracellular iron accumulation and eventual initiation of ferroptosis [41]. Additionally, the cystine/glutamate antiporter essential subunit, solute carrier family 7 member 11 (SLC7A11), is regulated by lncRNAs, thereby modulating ferroptosis. Notably, LINC00618 has been shown to interact with lymphoid-specific helicase(LSH), leading to the inhibition of SLC7A11 expression and the induction of ferroptosis [19].

Moreover, the intricate interplay between lncRNAs and the regulation of ferroptosis extends to their pivotal roles in its suppression. For example, lncRNA OIP5-AS1 has been demonstrated to attenuate ferroptosis in prostate cancer cells by promoting the upregulation of SLC7A11 expression via the silencing of miR-128-3P [42]. Within the antioxidant system, GPX4 assumes a central position, and lncRNAs exert their regulatory influence over ferroptosis by modulating GPX4 activity. Notably, LINC01134 has been shown to enhance GPX4 expression by facilitating the binding of nuclear factor NRF2 to the GPX4 promoter, thereby mitigating ferroptosis [43].

Furthermore, certain lncRNAs intricately regulate ferroptosis in cancer cells by modulating the protein expression levels of genes pivotal to ferroptosis. NRF2, a central regulator of oxidative stress response pathways, including the regulation of GPX4, also emerges as a key mediator of resistance to diverse chemotherapy agents and targeted therapeutics by impeding ferroptosis signaling cascades [44]. For instance, NRF2 functions dually to curtail ROS generation while concomitantly bolstering SLC7A11 expression, thereby restricting cellular iron uptake. The research elucidates that LINC00239 achieves inhibition of ferroptosis in colorectal cancer cells by stabilizing NRF2 through its interaction with Kelch-like ECH-associated protein 1 (Keap1) [45]. In summary, lncRNA plays indispensable roles throughout the entire process of ferroptosis development and occurrence.

**Fig. 2:**
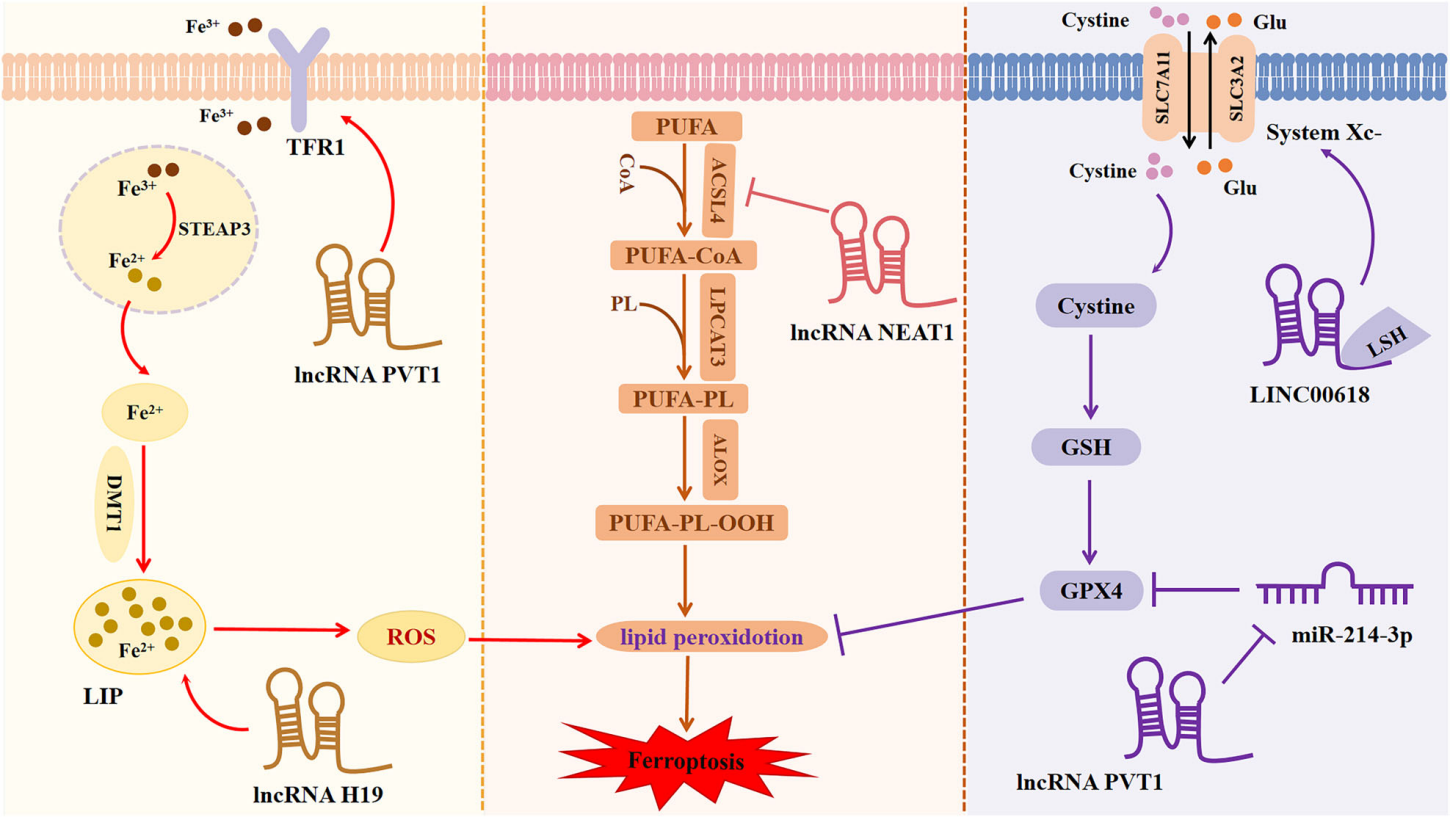
Regulation mechanisms of lncRNAs to ferroptosis. Left: Regulations of lncRNAs to iron metabolism. Middle: Regulations of lncRNAs to lipid metabolism. Right: Regulation of lncRNAs to amino acid metabolism.

In summary, the rapidly expanding field of lncRNA research has elucidated the intricate regulatory mechanisms through which these molecules orchestrate ferroptosis, thereby identifying novel avenues for disease therapeutics and underscoring the necessity for further investigation (Fig. 2).

## LncRNA and inflammation

In recent years, research has highlighted the significant regulatory role of lncRNA as crucial molecular modulators in inflammation. They exert this influence by modulating the expression of inflammatory genes and regulating the activation of inflammatory signaling pathways, thereby playing a pivotal influence over the onset and progression of inflammation.

### Role of lncRNAs in modulating pro-inflammatory cytokines

LncRNAs influence the production of pro-inflammatory cytokines and the activation status of immune cells by interacting with multiple molecular targets. TNF-α, a pro-inflammatory cytokine produced by T helper 1 (Th1) cells, is secreted by macrophages and exerts significant regulatory effects in various inflammatory conditions. LncRNA has been implicated in modulating the progression of inflammatory diseases by impacting TNF-α levels, as evidenced by research [46]. For instance, in colitis, there is an observed elevation in the expression of lncRNA DANCR. It has been demonstrated that attenuation of lncRNA DANCR expression leads to a reduction in TNF-α levels, thereby mitigating inflammation and suggesting as a therapeutic strategy for colitis [47]. IL-1 serves as a pivotal pro-inflammatory cytokine, while lncRNA HULC has been demonstrated to exert an inhibitory effect on IL-1 expression, consequently attenuating the inflammatory response [48]. Additionally, it has been elucidated that lncRNA NEAT1 functions to suppress the Toll-like receptor 2 (TLR2)/NF-κB signaling pathway, a pathway intricately involved in myocardial injury, wherein the activation of TLR2/NF-κB elicits an upregulation of IL-1. Thus, the modulation exerted by lncRNA NEAT1 on IL-1 levels may serves to ameliorate myocardial inflammation [49]. IL-4 is an inflammation factor of significant biological importance, upregulated in various inflammatory diseases [46]. One of its mechanisms in inflammation regulation involves inducing M2 polarization in macrophages. However, the overexpression of lncRNA PTPRE-AS1 can decrease IL-4 levels, thereby reducing M2 polarization in macrophages, potentially offering a therapeutic approach for pulmonary allergic inflammation [50]. Conversely, in a DSS-induced mouse colitis model, a deficiency in lncRNA PTPRE-AS1 promotes M2 macrophage activation, thus inhibiting the progression of colitis [50]. These findings suggest that lncRNA PTPRE-AS1 may serve as a crucial regulatory factor in other inflammatory diseases as well.

### Role of lncRNAs in modulating anti-inflammatory cytokines

In terms of anti-inflammatory responses, lncRNAs can inhibit excessive inflammation by regulating the expression of anti-inflammatory cytokines such as IL-10. IL-10, as a vital anti-inflammatory cytokine, plays a pivotal role in autoimmune and inflammatory disorders [51]. The regulatory influence of lncRNAs on IL-10 has significant implications for the progression of diverse inflammatory pathologies. Targeted modulation of lncRNAs to adjust IL-10 levels represents a promising avenue for therapeutic intervention in inflammatory diseases. Notably, studies have elucidated that silencing of lncRNA NEAT1 can augment IL-10 expression, thereby alleviating asthma-related inflammation [52]. Additionally, in the context of Alzheimer’s disease (AD) neuroinflammation, lncRNA MALAT1 downregulates the expression of IL-6 and TNF-α while concurrently enhancing IL-10 levels, suggesting the potential therapeutic value of targeting lncRNA MALAT1 in AD [53].

**Fig. 3:**
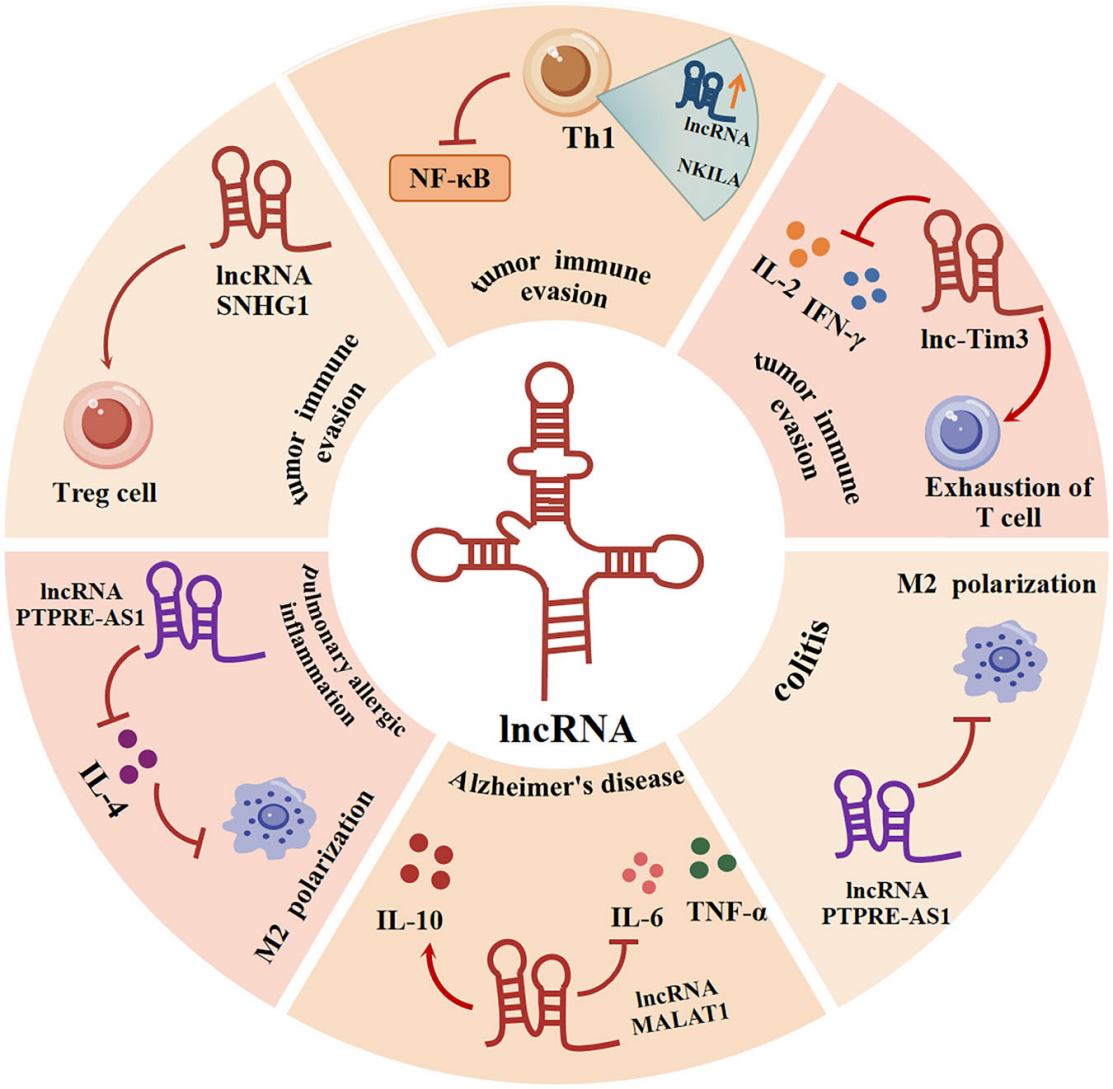
The crosstalk between lncRNA and inflammatory cytokines. LncRNAs regulate immune cells and inflammatory factors, thereby modulating tumor immune evasion, colitis, Alzheimer's disease, and pulmonary allergic inflammation. → indicates promotion, ⊥ indicates inhibition.

Overall, lncRNAs exert a regulatory influence on inflammation by modulating inflammatory mediators and signaling pathways. A comprehensive exploration of the functions and regulatory mechanisms of these lncRNAs is essential for unraveling the molecular intricacies of inflammation regulation, thereby potentially providing novel strategies for the treatment of associated diseases (Fig. 3).

## LncRNA and tumor immunity

Antigen presentation defects, T cell exhaustion, and the presence of tumor-associated macrophages (TAMs), etc., are key factors leading to tumor immune evasion and resistance to cancer therapy, these factors are also the primary focus for improving existing cancer immunotherapies [54]. Research has demonstrated the involvement of lncRNAs in multiple facets of cancer immunity, including antigen presentation to T cell exhaustion [55]. Dendritic cells (DCs) have the capability to process and present tumor antigens, thereby activating CD8^+^ T cells. Tumor-associated antigens can be captured and presented by DCs, with surface receptor molecules on CD8^+^ T cells selectively recognizing and binding to the major histocompatibility complex class I (MHC I). Subsequently, activated CD8^+^ T cells migrate to the tumor site, thereby identifying and eliminating cancer cells. Within DCs, it has been observed that the lncRNA DC can modulate the secretion of TNF-α, IL-6, and IFN-γ. Knockdown of lncRNA DC can reduce the differentiation and antigen-presenting capabilities of DCs. Antigen presentation is a crucial process for triggering effective immune responses. LncRNAs facilitate tumor immune evasion by dampening antigen presentation mechanisms [56]. Notably, lncRNA NKILA is highly expressed within cytotoxic T cells and Th1 cells, modulating tumor cell immune evasion by inhibiting NF-κB activation [57]. The majority of tumors exhibit significant infiltration by regulatory T cells (Tregs), a phenomenon that is associated with tumor immune evasion. Investigations suggest that lncRNA SNHG1 functions to hinder Tregs differentiation, consequently suppressing tumor immune evasion. IFN-γ is a multifunctional cytokine that plays a pivotal role in tumor immunity. Lnc-Tim3, upregulated in hepatocellular carcinoma patients, exerts inhibitory effects on the production of IFN-γ and IL-2 while promoting T-cell exhaustion, closely intertwining with anti-tumor immunity [58]. TAMs represent a pivotal constituent of the tumor microenvironment, exerting essential functions in the context of anti-tumor immunity. Relevant investigations underscore the notable implication of lncRNAs in TAM polarization. In essence, lncRNAs emerge as indispensable players in the orchestration of anti-tumor immune responses (Fig. 3).

## Role of lncRNAs in cancer progression via the ferroptosis pathway

### LncRNAs inhibit tumor progression through the ferroptosis pathway

Despite the availability of multiple pharmacological agents for the clinical management of tumors, the clinical efficacy remains suboptimal. Increasing evidence suggests that tumor cells have exhibited resistance to multiple chemotherapeutic agents, the 5-year survival rate among cancer patients is suboptimal. An increasing body of research evidence indicates that modulation of lncRNA expression can enhance the sensitivity of drug-resistant cancer cells to ferroptosis, thereby overcoming chemoresistance [59], underscoring their profound significance in this context. Studies have demonstrated that silencing the lncRNA HOTAIR can enhance the sensitivity of esophageal cancer (EC) cells to 5-fluorouracil (FU) chemotherapy [60]. According to reports, LINC00261 has also demonstrated regulatory effects on the chemosensitivity of EC cells to 5-FU, suggesting that targeting these lncRNAs in combination with chemotherapy agents may play a significant role in overcoming clinical drug resistance in EC [61]. The relevant evidence indicates that lncRNAs play a pivotal role in EC by regulating ferroptosis. For example, research demonstrates that lncRNA TMEM44-AS1 upregulates GPX4 expression via insulin-like growth factor 2 mRNA binding protein 2 (IGF2BP2), thereby inhibiting ferroptosis. This suggests that targeting lncRNA TMEM44-AS1 could serve as a potential therapeutic approach for combating esophageal squamous cell carcinoma (ESCC) [62]. Reportedly, the attenuation of lncRNA BBOX1-AS1 has been documented to decrease SLC7A11 levels, facilitating ferroptosis in ESCC cells and consequently impeding the advancement of ESCC [63]. The evidence indicates that ferroptosis-related lncRNAs may represent novel pharmacological targets for esophageal cancer, underscoring the importance of additional in-depth research.

Hepatocellular carcinoma (HCC) is one of the most common malignant tumors globally. Therefore, finding diagnostic markers and exploring new treatment methods are of great significance for treating liver cancer patients. Several studies have commenced investigating the regulatory role of lncRNAs in ferroptosis within HCC cells. The lncRNA HEPFAL mediates the reduction of SLC7A11 levels by promoting ubiquitination, consequently increasing ROS and iron levels, thereby facilitating ferroptosis in HCC [64]. The lncRNA NEAT1 has been demonstrated to participate in various forms of cell death. Notably, lncRNA NEAT1 has been demonstrated to be intimately associated with the process of ferroptosis. For example, investigations suggest that lncRNA NEAT1 can drive the upregulation of MIOX by interacting with miR-362-3p, thereby inducing ferroptosis in HCC upon exposure to Erastin and RSL3, thus impeding the progression of HCC [65]. Erastin upregulates the expression of lncRNA GABPB1 AS-1, resulting in the inhibition of GABPB1 translation and subsequent reduction in peroxiredoxin 5 (PRDX5) expression. Consequently, this facilitates the accumulation of ROS and ultimately induces ferroptosis in HepG2 cells [66]. Emerging research indicates that lncRNAs exert regulatory control over ferroptosis through intricate interactions with miRNAs. For example, lncRNA PVT1 has been demonstrated to impede GPX4 expression by inhibiting the translation of miR-214-3p, thereby inducing ferroptosis in HCC cells [67]. These lncRNAs hold promise as plausible diagnostic modalities for early detection of HCC and may serve as prospective therapeutic targets.

Numerous preclinical studies have demonstrated that lncRNAs associated with ferroptosis exhibit varying expression levels across different tumor cells and tissues, which not only suggests the potential of lncRNAs as diagnostic and prognostic biomarkers [68] but also offers a novel perspective for personalized cancer treatment [69]. The widespread observation of abnormal expression of lncRNAs in breast cancer indicates that certain lncRNAs may serve as diagnostic biomarkers or potential therapeutic targets for the disease. Studies have identified that lncRNA FASA interacts with the Ahpc-TSA domain of PRDX1, thereby promoting the liquid-liquid phase separation of PRDX1, which leads to the accumulation of peroxides. This accumulation subsequently heightens the susceptibility of triple-negative breast cancer (TNBC) to ferroptosis [70]. LncRNA P53RRA not only inhibits the progression of lung cancer but also suppresses the development of breast cancer by promoting ferroptosis [71]. Other lncRNAs associated with ferroptosis have been discovered, potentially offering promising therapeutic targets for breast cancer. Furthermore, the bioinformatic analysis of breast cancer biomarkers plays a pivotal role in predicting prognosis and enhancing treatment outcomes [72]. Evidence suggests that Erastin upregulates the expression of lncRNA A2M-AS1, which in turn interacts directly with poly(C)-binding protein 3 (PCBP3), consequently facilitating the occurrence of ferroptosis in pancreatic cancer cells [73].

**Fig. 4:**
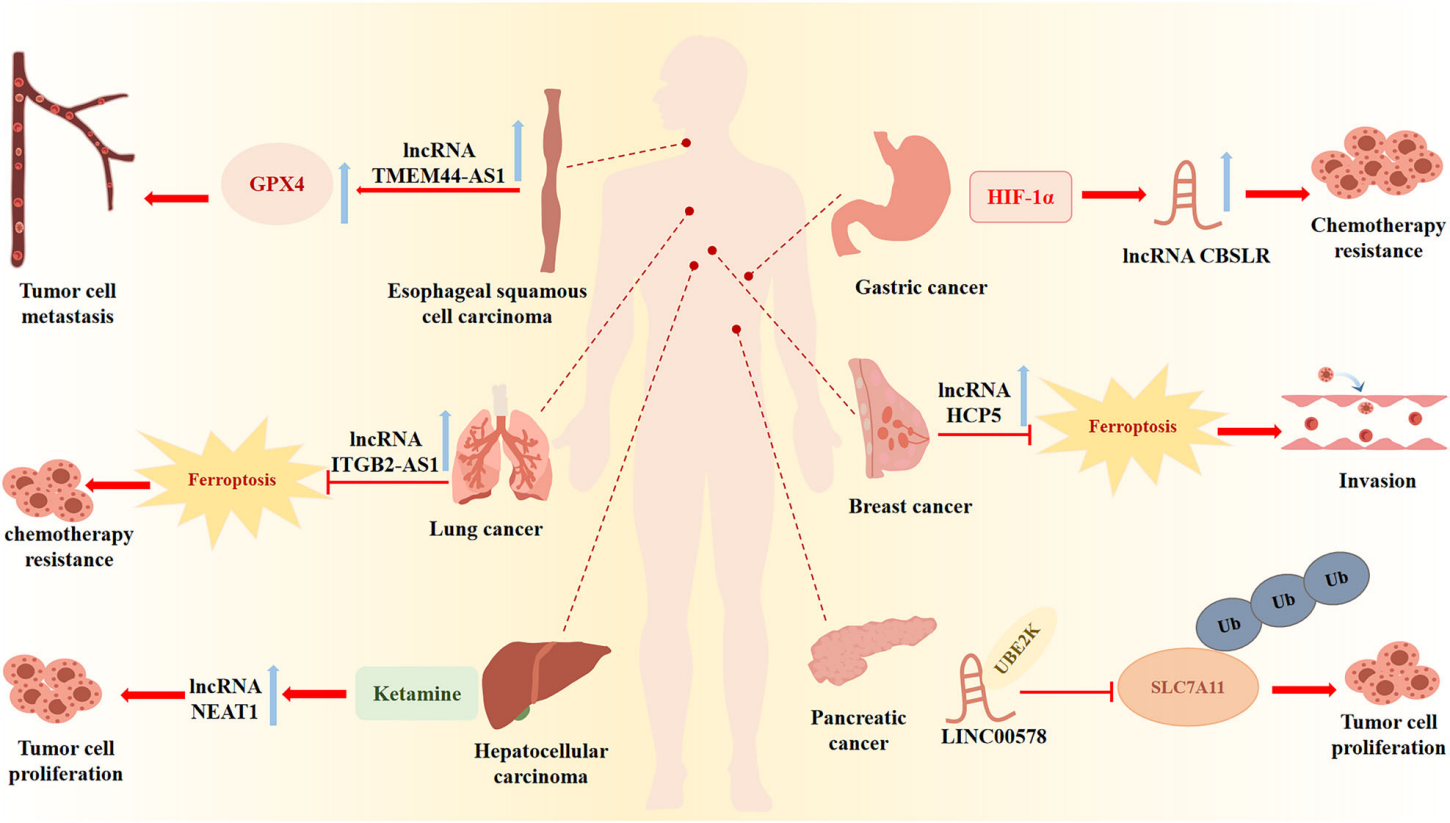
Long non-coding RNAs play pivotal roles in the pathogenesis and progression of diverse cancer types. The expression of lncRNAs and their modulation of the ferroptosis pathway collectively regulate key processes such astumor cell proliferation, invasion, and migration. These regulatory actions significantly influence the initiation andprogression of tumor development. → indicates promotion and increased expression, ⊥ indicates inhibition.

In conclusion, lncRNAs have been demonstrated to impede cancer progression by regulating ferroptosis. This regulation can shift the cellular equilibrium in favor of ferroptosis cells. LncRNAs offer a novel approach to cancer therapy by modulating these regulatory mechanisms, rendering them prospective therapeutic targets for the selective induction of ferroptosis in cancer cells. However, the specific mechanisms by which lncRNAs influence cancer treatment through the regulation of ferroptosis have not been adequately investigated in clinical trials, and further exploration is warranted in this field. Moreover, considering the heterogeneity of lncRNA expression across different cancers, the prospects for the application of targeted lncRNA therapeutic strategies in personalized medicine are promising (Fig. 4) (Table 1).

**Table 1:** Examples of lncRNAs regulations ferroptosis in cancer.

Name	Role in ferroptosis	Mechanism of action	Reference
LncRNA HEPFAL	Induces ferroptosis in HCC	Promoting the ubiquitination of SLC7A11	[64]
LncRNA PVT1	Inhibits ferroptosis in HCC	Sponges miR-214-3p, upregulates GPX4	[67]
LncRNA GABPB1 AS-1	Induces ferroptosis in HCC	Inhibits GABPB1 translation, downregulates GABPB1 and PRDX5	[66]
LINC00618	Induces ferroptosis in leukemias	Downregulates SLC7A11 via attenuation of LSH expression	[19]
LncRNA FASA	Induces ferroptosis in TNBC	Enhancing the formation of PRDX1	[70]
LncRNA CASC2	Induces ferroptosis in Gastric cancer	Downregulates SLC7A11	[74]
LncRNA MT1DP	Induces ferroptosis in NSCLC	Stabilizes miR-365a-3p, downregulates NRF2	[75]
LncRNA TMEM44-AS1	Inhibits ferroptosis in ESCC	Upregulates GPX4	[62]
LncRNA BBOX1-AS1	Inhibits ferroptosis in ESCC	Upregulates SLC7A11	[63]
LncRNA HCP5	Inhibits ferroptosis in TNBC	Upregulates GPX4	[76]
LINC00578	Inhibits ferroptosis in Pancreatic cancer	Inhibition of SLC7A11 ubiquitination	[77]
LINC01133	Inhibits ferroptosis in Pancreatic cancer	Stabilizes FSP1	[78]
LncRNA CBSLR	Inhibits ferroptosis in Gastric cancer	Downregulates ACSL4	[79]
LncRNA BDNF-AS	Inhibits ferroptosis in Gastric cancer	Upregulates VDAC3	[80]
LncRNA ITGB2-AS1	Inhibits ferroptosis in NSCLC	Inhibition of p53 expression	[81]

*HCC* Hepatocellular carcinoma, TNBC Triple-negative breast cancer, NSCLC non-small cell lung cancer, ESCC Esophageal squamous cell carcinoma.

### LncRNAs promote tumor progression through the ferroptosis pathway

The regulation of ferroptosis by lncRNAs is a complex process. The expression levels of ferroptosis-associated lncRNAs vary across different stages of cancer in patients, indicating their potential utility as biomarkers for monitoring disease progression and prognosis. Several ferroptosis-related lncRNAs have been substantiated to be associated with the prognostic outcomes of patients with lung carcinoma, exemplified by lncRNA PELATON [74]. Drug resistance is the main reason for the poor clinical treatment outcomes of cancer, with a discernible correlation established between the heightened expression of lncRNAs and the acquisition of chemoresistance in lung carcinoma. Platinum resistance poses a primary challenge in the clinical management of non-small cell lung cancer (NSCLC). The lncRNA ITGB2-AS1, through its interaction with FOS-like 2(FOSL2), enhances nicotinamide phosphoribosyl transferase (NAMPT) expression, thereby further suppressing p53-mediated ferroptosis, and consequently fostering cisplatin resistance in NSCLC [75]. Therefore, the regulatory role of lncRNA in ferroptosis may contribute to a better understanding of drug resistance mechanisms in lung cancer. Targeting these ferroptosis-associated lncRNAs holds promise as a promising approach for treating lung cancer. Additionally, lncRNAs also play a significant role in breast cancer. For instance, findings suggest that HCP5-132aa, encoded by lncRNA HCP5, exerts a suppressive effect on ferroptosis while promoting the malignant progression of TNBC. This effect is achieved through the upregulation of GPX4 expression and the concurrent reduction of lipid reactive ROS levels [76]. An increasing body of research indicates that lncRNAs exert considerable influence on the initiation and progression of pancreatic cancer. The findings suggest that LINC00578 mediates the inhibition of ferroptosis in pancreatic cancer by specifically interacting with UBE2K, thereby impeding the ubiquitination process of SLC7A11, and consequently fostering the progression of pancreatic cancer [77]. As reported, LINC01133 augments the stability of the ferroptosis suppressor FSP1 by facilitating the formation of a LINC01133-FUS-FSP1 complex. This interaction subsequently impedes ferroptosis, thereby fostering the progression of pancreatic cancer [78]. These lncRNAs facilitate the pathological advancement of pancreatic cancer by inhibiting ferroptosis in cancer cells. Conversely, the suppression of these lncRNAs’ expression could promote ferroptosis and thereby inhibit the progression of pancreatic cancer. These findings may provide novel research directions for exploring the specific regulatory mechanisms between ferroptosis-related lncRNAs and pancreatic cancer.

Meanwhile, ferroptosis-associated lncRNAs play a pivotal role in the pathogenesis of gastric cancer. For instance, the lncRNA CASC2 exerts its influence by downregulating SLC7A11 expression via SOCS2, thereby promoting ferroptosis in gastric cancer cells [79]. The hypoxic microenvironment has been proven to be correlated with tumor drug resistance and poor prognosis. The hypoxia-inducible lncRNA CBSLR has been shown to notably increase the expression levels of acyl-CoA synthetase long-chain family member 4 (ACSL4) upon its knockdown. This subsequently regulates ferroptosis in gastric cancer cells, suggesting that lncRNA CBSLR may potentially be a therapeutic target for hypoxic tumors [80]. The lncRNA BDNF-AS/WDR5/FBXW7 axis modulates ferroptosis in gastric cancer by impacting the ubiquitination of voltage-dependent anion channel 3 (VDAC3), thereby mediating peritoneal metastasis of gastric cancer [81].

The results of these studies collectively indicate that as more lncRNAs associated with ferroptosis are identified and studied, they are likely to become important therapeutic targets for tumors in the future. However, the instability of lncRNAs leads to low delivery efficiency, and they face challenges in clinical application, including tolerability, off-target effects, and potential immune responses [82], further research is required to elucidate the precise regulatory functions of lncRNAs in cancer via ferroptosis, to develop more efficacious clinical treatments. Future studies that encapsulate anticancer drugs (such as sorafenib) and ferroptosis-related lncRNAs within nanoparticles for cancer treatment could significantly enhance the clinical application potential of lncRNA-based therapies in oncology, representing a promising novel approach to cancer treatment (Fig. 4) (Table 1).

## Therapeutic potential of lncRNA in ferroptosis

In the current landscape of oncological research, the exploration of lncRNAs for cancer therapeutics is still in its nascent phase. The development of lncRNA-based therapeutics is predominantly constrained by challenges pertaining to specificity, delivery mechanisms, and patient tolerance. Therefore, investigators are innovating new lncRNA therapeutic strategies to address these shortcomings and augment the feasibility of clinical translation. For instance, research has indicated that lncRNA MT1DP, encapsulated within folate-modified liposomes, modulates the miR-365a-3p/NRF2 axis in non-small cell lung cancer (NSCLC), thereby potentiating the ferroptotic response induced by Erastin and facilitating ferroptosis in lung cancer cells [83]. This study suggests that the combination of nanoparticles and lncRNA for the targeted delivery to specific cancer cells represents a highly promising strategy with significant potential for clinical applications in the treatment of lung cancer. Despite the promising advancements in using lncRNAs for cancer therapeutics, the clinical application of lncRNA-mediated ferroptosis faces considerable challenges. First of all, validating the functional attributes and therapeutic potential of lncRNAs in vivo presents a formidable challenge, particularly in light of the low homology observed across different species. Although a subset of human-mouse homologous lncRNAs has been identified, a significant number of human lncRNAs have eluded detection in murine models. Consequently, the direct translation of therapeutic modalities from in vitro and animal models to human applications may be difficult and necessitates further evaluation. Moreover, the mechanisms regulating ferroptosis by lncRNAs are not yet fully understood, limiting their application in modulating ferroptosis. Consequently, further research is essential to overcome these limitations.

LncRNAs implicated in the regulation of ferroptosis have exhibited significant therapeutic efficacy, thereby underscoring their considerable promise in the amelioration of a spectrum of other pathological conditions. As an illustration, lncRNA ZFAS1 exerts a critical modulatory effect on the pathophysiological trajectory of dilated cardiomyopathy (DCM) [84]. It does so by antagonizing the ferroptotic process within cardiomyocytes, thereby holding substantial implications for disease progression and offering a potential target for therapeutic intervention.

It is of particular academic interest that due to their distinctive ability to facilitate the transport of biological molecules, the delivery of lncRNAs through exosomes has emerged as a novel and promising therapeutic avenue. Exosomal lncRNAs associated with the phenomenon of ferroptosis have been empirically shown to exert a multifaceted influence on a range of pathological states. This includes, but is not limited to, atherosclerosis, cardiovascular disorders, diabetes mellitus, and neoplastic conditions, thereby highlighting the intricate regulatory roles these molecules play in the pathogenesis of these diseases [85]. Furthermore, Huang et al. demonstrated that atorvastatin-pretreated mesenchymal stem cells (MSCs) facilitate angiogenesis and cardiomyocyte survival in myocardial infarction (MI) hearts by upregulating lncRNA H19 and its release via exosomes [86]. The potential of exosomal lncRNAs in the therapeutic context of ferroptosis is underscored by their ability to precisely target and deliver these molecules to specific cellular recipients, subsequently modulating gene expression and thereby introducing for innovative therapeutic strategies against diseases. Despite the ongoing challenges in the effective drug delivery of therapeutics within the realm of bioengineering, research in this domain is still in its nascent phase. However, the relentless pursuit of innovation holds the promise of delivering precise and personalized treatment modalities for a multitude of pathologies, which could potentially transform the therapeutic paradigm in the future [87].

Despite the nascent state of research on lncRNAs in the context of cancer therapy and the paucity of clinical data, a review of the ClinicalTrials.gov database has identified several noteworthy developments in ongoing clinical trials. In particular, the NCT05708209 trial has been concluded with the objective of evaluating the accuracy of lncRNA MALAT1 as a potential diagnostic biomarker for oral squamous cell carcinoma. The NCT05334849 trial has also reached its conclusion, confirming the potential of circulating exosomal lncRNA-GC1 as a GC-specific biomarker. Similarly, the NCT04767750 trial has been completed, with the objective of exploring the role of lncRNA H19 in the regulation of IGF-1R expression and investigating the mechanistic links between HCC and type 2 diabetes mellitus (T2DM). Additionally, the NCT06534242 trial has been concluded with the objective of investigating the correlation between LINC00511 and the susceptibility to, as well as the pathogenesis of, colorectal cancer. Moreover, the status of the NCT03469544 trial remains uncertain. The study aimed to validate lncRNA HOTAIR as a diagnostic biomarker for thyroid cancer. Although these studies have provided novel insights into the clinical application of lncRNAs in various diseases, there is currently a lack of clinical research data regarding the regulation of ferroptosis by lncRNAs as a therapeutic approach for cancer treatment.

## Summary and prospects

In this review, our objective is to delineate the roles of ferroptosis and lncRNAs in modulating inflammatory responses and tumor progression, with a particular emphasis on the significant potential of ferroptosis-associated lncRNAs in cancer therapy and diagnostics. The aim of this paper is to offer critical insights into the clinical applications of targeting ferroptosis-related lncRNAs in tumor immunotherapy.

In summary, ferroptosis has demonstrated significant potential as a therapeutic strategy for cancer. Ferroptosis inducers facilitate the ferroptotic death of tumor cells, thereby inhibiting tumor progression. Moreover, IFN-γ produced by activated T cells, when combined with specific fatty acids in the tumor microenvironment, can also induce ferroptosis in tumor cells. This not only indicates that ferroptosis can directly lead to tumor cell death but also enhances the antitumor effects of the immune system by altering the tumor microenvironment. Furthermore, the immune system’s promotion of ferroptosis to kill tumor cells reveals a novel mechanism for inhibiting tumor growth. Notably, the combination of nanomedicine-based ferroptosis inducers with immune checkpoint blockade therapy enhances the sensitivity of cancer cells to ferroptosis, suggesting that the integration of immunotherapy with ferroptosis induction is a highly promising treatment approach. While ferroptosis can significantly augment tumor immunotherapy, ferroptosis inducers may compromise the survival of T cells, resulting in reduced anti-tumor immune functionality. The clinical feasibility of ferroptosis in cancer therapy still faces several unresolved issues. On one hand, the challenge remains to promote cancer cell ferroptosis without inducing ferroptosis in immune cells. On the other hand, there is concern regarding crosstalk between diseases, while inducing ferroptosis with GPX4 inhibitors, there have been observed impacts on neurological functionality.

Ferroptosis is regarded as a promising therapeutic target for inflammatory disorders, however, there is currently a deficiency in discerning ferroptosis biomarkers tailored to specific inflammatory pathologies. The identification of pertinent ferroptosis biomarkers could significantly improve the therapeutic efficacy across a range of inflammatory ailments. Although preclinical investigations have demonstrated pronounced mitigation of inflammatory disorders through ferroptosis inhibition in diverse animal models, the clinical landscape remains relatively devoid of comprehensive data. Thus, there is an urgent need for clinical trials to confirm the feasibility of inhibiting ferroptosis for improving the clinical outcomes of inflammatory diseases.

The potential of long non-coding RNAs (lncRNAs) as therapeutic targets in oncology has been substantiated by their multifaceted roles. Nonetheless, the application of lncRNAs as therapeutic targets is fraught with challenges, particularly concerning the targeting of lncRNAs and the efficient and secure delivery of therapeutic agents. The development of innovative delivery mechanisms is essential to ensure the precise and potent delivery of therapeutics to the intended lncRNAs. For instance, drug delivery systems based on extracellular vesicles, such as those utilizing exosome-based nanoparticles, may offer improved targeting for lncRNA therapies. Attaining this level of precision is paramount for optimizing therapeutic outcomes. Moreover, recent evidence has highlighted lncRNAs as promising candidates for cancer immunotherapy. Studies have revealed that lncRNAs associated with immunological ferroptosis are specific regulators of gene expression in immune cells, influencing both immune stimulation and suppression. This suggests that lncRNA targeting could potentially augment the efficacy of immunotherapies. Despite the promise, clinical research on lncRNAs related to ferroptosis remains limited. However, a deeper exploration of their functions and regulatory mechanisms within the immunological context is an emerging field with considerable potential. Such research is instrumental for unraveling the intricacies of antitumor immune modulation, understanding the pathogenesis of autoimmune diseases, and pinpointing prospective therapeutic targets.

## References

[1] Dixon SJ, Lemberg KM, Lamprecht MR, Skouta R, Zaitsev EM, Gleason CE, et al. Ferroptosis: an iron-dependent form of nonapoptotic cell death. Cell. 2012;149:1060–72. 22632970 10.1016/j.cell.2012.03.042 PMC3367386

[2] Yagoda N, von Rechenberg M, Zaganjor E, Bauer AJ, Yang WS, Fridman DJ, et al. RAS-RAF-MEK-dependent oxidative cell death involving voltage-dependent anion channels. Nature. 2007;447:864–8. 17568748 10.1038/nature05859 PMC3047570

[3] Yang WS, Stockwell BR. Synthetic lethal screening identifies compounds activating iron-dependent, nonapoptotic cell death in oncogenic-RAS-harboring cancer cells. Chem Biol. 2008;15:234–45. 18355723 10.1016/j.chembiol.2008.02.010 PMC2683762

[4] Tang D, Chen X, Kang R, Kroemer G. Ferroptosis: molecular mechanisms and health implications. Cell Res. 2021;31:107–25. 33268902 10.1038/s41422-020-00441-1 PMC8026611

[5] Friedmann Angeli JP, Krysko DV, Conrad M. Ferroptosis at the crossroads of cancer-acquired drug resistance and immune evasion. Nat Rev Cancer. 2019;19:405–14. 31101865 10.1038/s41568-019-0149-1

[6] Li B, Yang L, Peng X, Fan Q, Wei S, Yang S, et al. Emerging mechanisms and applications of ferroptosis in the treatment of resistant cancers. Biomedicine Pharmacother = Biomedecine Pharmacotherapie. 2020;130:110710. 10.1016/j.biopha.2020.110710 33568263

[7] Wu Y, Yu C, Luo M, Cen C, Qiu J, Zhang S, et al. Ferroptosis in Cancer Treatment: Another Way to Rome. Front Oncol. 2020;10:571127. 33102227 10.3389/fonc.2020.571127 PMC7546896

[8] Wapinski O, Chang HY. Long noncoding RNAs and human disease. Trends cell Biol. 2011;21:354–61. 21550244 10.1016/j.tcb.2011.04.001

[9] Whitehead J, Pandey GK, Kanduri C. Regulation of the mammalian epigenome by long noncoding RNAs. Biochimica et Biophysica acta. 2009;1790:936–47. 19015002 10.1016/j.bbagen.2008.10.007

[10] Chen DL, Lu YX, Zhang JX, Wei XL, Wang F, Zeng ZL, et al. Long non-coding RNA UICLM promotes colorectal cancer liver metastasis by acting as a ceRNA for microRNA-215 to regulate ZEB2 expression. Theranostics. 2017;7:4836–49. 29187907 10.7150/thno.20942 PMC5706103

[11] Wang M, Mao C, Ouyang L, Liu Y, Lai W, Liu N, et al. Long noncoding RNA LINC00336 inhibits ferroptosis in lung cancer by functioning as a competing endogenous RNA. Cell Death Differ. 2019;26:2329–43. 30787392 10.1038/s41418-019-0304-y PMC6889193

[12] Choi SW, Kim HW, Nam JW. The small peptide world in long noncoding RNAs. Brief Bioinforma. 2019;20:1853–64. 10.1093/bib/bby055 PMC6917221 30010717

[13] Zhang Y, Wang X, Hu C, Yi H. Shiny transcriptional junk: lncRNA-derived peptides in cancers and immune responses. Life Sci. 2023;316:121434. 36706831 10.1016/j.lfs.2023.121434

[14] Zhou B, Yang H, Yang C, Bao YL, Yang SM, Liu J, et al. Translation of noncoding RNAs and cancer. Cancer Lett. 2021;497:89–99. 33038492 10.1016/j.canlet.2020.10.002

[15] Zuckerman B, Ulitsky I. Predictive models of subcellular localization of long RNAs. RNA (N. Y, NY). 2019;25:557–72. 10.1261/rna.068288.118 PMC6467007 30745363

[16] Taheri M, Badrlou E, Hussen BM, Kashi AH, Ghafouri-Fard S, Baniahmad A. Importance of long non-coding RNAs in the pathogenesis, diagnosis, and treatment of prostate cancer. Front Oncol. 2023;13:1123101. 37025585 10.3389/fonc.2023.1123101 PMC10070735

[17] Hao L, Wu W, Xu Y, Chen Y, Meng C, Yun J. et al. LncRNA-MALAT1: a key participant in the occurrence and development of cancer. Molecules. 2023;28(2126):28 10.3390/molecules28052126 PMC10004581 36903369

[18] Hou J, Zhang G, Wang X, Wang Y, Wang K. Functions and mechanisms of lncRNA MALAT1 in cancer chemotherapy resistance. Biomark Res. 2023;11:23. 36829256 10.1186/s40364-023-00467-8 PMC9960193

[19] Wang Z, Chen X, Liu N, Shi Y, Liu Y, Ouyang L, et al. A Nuclear Long Non-Coding RNA LINC00618 Accelerates Ferroptosis in a Manner Dependent upon Apoptosis. Mol Ther : J Am Soc Gene Ther. 2021;29:263–74. 10.1016/j.ymthe.2020.09.024 PMC7791008 33002417

[20] Yang K, Zeng L, Ge A, Wang S, Zeng J, Yuan X, et al. A systematic review of the research progress of non-coding RNA in neuroinflammation and immune regulation in cerebral infarction/ischemia-reperfusion injury. Front Immunol. 2022;13:930171. 36275741 10.3389/fimmu.2022.930171 PMC9585453

[21] Ye L, Wen X, Qin J, Zhang X, Wang Y, Wang Z, et al. Metabolism-regulated ferroptosis in cancer progression and therapy. Cell Death Dis. 2024;15:196. 38459004 10.1038/s41419-024-06584-y PMC10923903

[22] Li W, Liang L, Liu S, Yi H, Zhou Y. FSP1: a key regulator of ferroptosis. Trends Mol Med. 2023;29:753–64. 37357101 10.1016/j.molmed.2023.05.013

[23] Mao C, Liu X, Zhang Y, Lei G, Yan Y, Lee H, et al. DHODH-mediated ferroptosis defence is a targetable vulnerability in cancer. Nature. 2021;593:586–90. 33981038 10.1038/s41586-021-03539-7 PMC8895686

[24] Yeung YT, Aziz F, Guerrero-Castilla A, Arguelles S. Signaling Pathways in Inflammation and Anti-inflammatory Therapies. Curr Pharm Des. 2018;24:1449–84. 29589535 10.2174/1381612824666180327165604

[25] Li D, Wu M. Pattern recognition receptors in health and diseases. Signal Transduct Target Ther. 2021;6:291. 34344870 10.1038/s41392-021-00687-0 PMC8333067

[26] Deng L, He S, Guo N, Tian W, Zhang W, Luo L. Molecular mechanisms of ferroptosis and relevance to inflammation. Inflamm Res : J Eur Histamine Res Soc [et al]. 2023;72:281–99. 10.1007/s00011-022-01672-1 PMC9762665 36536250

[27] Chen X, Kang R, Kroemer G, Tang D. Ferroptosis in infection, inflammation, and immunity. J. Exp. Med. 2021;218:e20210518. 33978684 10.1084/jem.20210518 PMC8126980

[28] Kim S, Keku TO, Martin C, Galanko J, Woosley JT, Schroeder JC, et al. Circulating levels of inflammatory cytokines and risk of colorectal adenomas. Cancer Res. 2008;68:323–8. 18172326 10.1158/0008-5472.CAN-07-2924 PMC2675825

[29] Mitchell JP, Carmody RJ. NF-κB and the Transcriptional Control of Inflammation. Int Rev cell Mol Biol. 2018;335:41–84. 29305014 10.1016/bs.ircmb.2017.07.007

[30] Yan N, Xu Z, Qu C, Zhang J. Dimethyl fumarate improves cognitive deficits in chronic cerebral hypoperfusion rats by alleviating inflammation, oxidative stress, and ferroptosis via NRF2/ARE/NF-κB signal pathway. Int Immunopharmacol. 2021;98:107844. 34153667 10.1016/j.intimp.2021.107844

[31] Gupta U, Ghosh S, Wallace CT, Shang P, Xin Y, Nair AP, et al. Increased LCN2 (lipocalin 2) in the RPE decreases autophagy and activates inflammasome-ferroptosis processes in a mouse model of dry AMD. Autophagy. 2023;19:92–111. 35473441 10.1080/15548627.2022.2062887 PMC9809950

[32] Meihe L, Shan G, Minchao K, Xiaoling W, Peng A, Xili W, et al. The Ferroptosis-NLRP1 Inflammasome: The Vicious Cycle of an Adverse Pregnancy. Front cell Developmental Biol. 2021;9:707959. 10.3389/fcell.2021.707959 PMC8417576 34490257

[33] Chen Y, Fang ZM, Yi X, Wei X, Jiang DS. The interaction between ferroptosis and inflammatory signaling pathways. Cell Death Dis. 2023;14:205. 36944609 10.1038/s41419-023-05716-0 PMC10030804

[34] Matsushita M, Freigang S, Schneider C, Conrad M, Bornkamm GW, Kopf M. T cell lipid peroxidation induces ferroptosis and prevents immunity to infection. J Exp Med. 2015;212:555–68. 25824823 10.1084/jem.20140857 PMC4387287

[35] Kang R, Chen R, Zhang Q, Hou W, Wu S, Cao L, et al. HMGB1 in health and disease. Mol Asp Med. 2014;40:1–116. 10.1016/j.mam.2014.05.001 PMC4254084 25010388

[36] Lv DJ, Song XL, Huang B, Yu YZ, Shu FP, Wang C, et al. HMGB1 Promotes Prostate Cancer Development and Metastasis by Interacting with Brahma-Related Gene 1 and Activating the Akt Signaling Pathway. Theranostics. 2019;9:5166–82. 31410208 10.7150/thno.33972 PMC6691575

[37] Efimova I, Catanzaro E, Van der Meeren L, Turubanova VD, Hammad H, Mishchenko TA. et al. Vaccination with early ferroptotic cancer cells induces efficient antitumor immunity. J Immunother Cancer. 2020;8:1869. 10.1136/jitc-2020-001369 PMC7668384 33188036

[38] Wang W, Green M, Choi JE, Gijón M, Kennedy PD, Johnson JK, et al. CD8(+) T cells regulate tumour ferroptosis during cancer immunotherapy. Nature. 2019;569:270–4. 31043744 10.1038/s41586-019-1170-y PMC6533917

[39] Zitvogel L, Kroemer G. Interferon-γ induces cancer cell ferroptosis. Cell Res. 2019;29:692–3. 31160718 10.1038/s41422-019-0186-z PMC6796847

[40] Lang X, Green MD, Wang W, Yu J, Choi JE, Jiang L, et al. Radiotherapy and Immunotherapy Promote Tumoral Lipid Oxidation and Ferroptosis via Synergistic Repression of SLC7A11. Cancer Discov. 2019;9:1673–85. 31554642 10.1158/2159-8290.CD-19-0338 PMC6891128

[41] Luo W, Wang J, Xu W, Ma C, Wan F, Huang Y, et al. LncRNA RP11-89 facilitates tumorigenesis and ferroptosis resistance through PROM2-activated iron export by sponging miR-129-5p in bladder cancer. Cell Death Dis. 2021;12:1043. 34728613 10.1038/s41419-021-04296-1 PMC8563982

[42] Zhang Y, Guo S, Wang S, Li X, Hou D, Li H, et al. LncRNA OIP5-AS1 inhibits ferroptosis in prostate cancer with long-term cadmium exposure through miR-128-3p/SLC7A11 signaling. Ecotoxicol Environ Saf. 2021;220:112376. 34051661 10.1016/j.ecoenv.2021.112376

[43] Kang X, Huo Y, Jia S, He F, Li H, Zhou Q, et al. Silenced LINC01134 Enhances Oxaliplatin Sensitivity by Facilitating Ferroptosis Through GPX4 in Hepatocarcinoma. Front Oncol. 2022;12:939605. 35875091 10.3389/fonc.2022.939605 PMC9304856

[44] Dodson M, Castro-Portuguez R, Zhang DD. NRF2 plays a critical role in mitigating lipid peroxidation and ferroptosis. Redox Biol. 2019;23:101107. 30692038 10.1016/j.redox.2019.101107 PMC6859567

[45] Han Y, Gao X, Wu N, Jin Y, Zhou H, Wang W, et al. Long noncoding RNA LINC00239 inhibits ferroptosis in colorectal cancer by binding to Keap1 to stabilize Nrf2. Cell Death Dis. 2022;13:742. 36038548 10.1038/s41419-022-05192-y PMC9424287

[46] Ashrafizadeh M, Zarrabi A, Mostafavi E, Aref AR, Sethi G, Wang L, et al. Non-coding RNA-based regulation of inflammation. Semin Immunol. 2022;59:101606. 35691882 10.1016/j.smim.2022.101606

[47] Zhang X, Ma L, Zhang C, Hou B, Zhou Y, Yu S. Silencing LncRNA-DANCR attenuates inflammation and DSS-induced endothelial injury through miR-125b-5p. Gastroenterologia y Hepatologia. 2021;44:644–53. 33317921 10.1016/j.gastrohep.2020.10.008

[48] Chen ZL, Chen YX, Zhou J, Li Y, Gong CY, Wang XB. LncRNA HULC alleviates HUVEC inflammation and improves angiogenesis after myocardial infarction through down-regulating miR-29b. Eur Rev Med Pharmacol Sci. 2020;24:6288–98. 32572926 10.26355/eurrev_202006_21527

[49] Wang SM, Liu GQ, Xian HB, Si JL, Qi SX, Yu YP. LncRNA NEAT1 alleviates sepsis-induced myocardial injury by regulating the TLR2/NF-κB signaling pathway. Eur Rev Med Pharmacol Sci. 2019;23:4898–907. 31210324 10.26355/eurrev_201906_18078

[50] Han X, Huang S, Xue P, Fu J, Liu L, Zhang C, et al. LncRNA PTPRE-AS1 modulates M2 macrophage activation and inflammatory diseases by epigenetic promotion of PTPRE. Sci Adv. 2019;5:eaax9230. 31844669 10.1126/sciadv.aax9230 PMC6905863

[51] Zheng Z, Huang G, Gao T, Huang T, Zou M, Zou Y, et al. Epigenetic Changes Associated With Interleukin-10. Front Immunol. 2020;11:1105. 32582189 10.3389/fimmu.2020.01105 PMC7287023

[52] Duan XJ, Zhang X, Ding N, Zhang JY, Chen YP. LncRNA NEAT1 regulates MMP-16 by targeting miR-200a/b to aggravate inflammation in asthma. Autoimmunity. 2021;54:439–49. 34448644 10.1080/08916934.2021.1966769

[53] Shi YL, Wang Q, Wei JC. Influence of lncRNA-MALAT1 on neuronal apoptosis in rats with cerebral infarction through regulating the ERK/MAPK signaling pathway. Eur Rev Med Pharmacol Sci. 2019;23:8039–48. 31599429 10.26355/eurrev_201909_19020

[54] Hu Q, Egranov SD, Lin C, Yang L. Long noncoding RNA loss in immune suppression in cancer. Pharmacol Therapeutics. 2020;213:107591. 10.1016/j.pharmthera.2020.107591 PMC7434710 32473960

[55] Wu M, Fu P, Qu L, Liu J, Lin A. Long Noncoding RNAs, New Critical Regulators in Cancer Immunity. Front Oncol. 2020;10:550987. 33194608 10.3389/fonc.2020.550987 PMC7662117

[56] Peltier DC, Roberts A, Reddy P. LNCing RNA to immunity. Trends Immunol. 2022;43:478–95. 35501219 10.1016/j.it.2022.04.002 PMC9647660

[57] Huang D, Chen J, Yang L, Ouyang Q, Li J, Lao L, et al. NKILA lncRNA promotes tumor immune evasion by sensitizing T cells to activation-induced cell death. Nat Immunol. 2018;19:1112–25. 30224822 10.1038/s41590-018-0207-y

[58] Ji J, Yin Y, Ju H, Xu X, Liu W, Fu Q, et al. Long non-coding RNA Lnc-Tim3 exacerbates CD8 T cell exhaustion via binding to Tim-3 and inducing nuclear translocation of Bat3 in HCC. Cell Death Dis. 2018;9:478. 29706626 10.1038/s41419-018-0528-7 PMC5924754

[59] Wang H, Fleishman JS, Cheng S, Wang W, Wu F, Wang Y, et al. Epigenetic modification of ferroptosis by non-coding RNAs in cancer drug resistance. Mol Cancer. 2024;23:177. 39192329 10.1186/s12943-024-02088-7 PMC11348582

[60] Zhang S, Zheng F, Zhang L, Huang Z, Huang X, Pan Z, et al. LncRNA HOTAIR-mediated MTHFR methylation inhibits 5-fluorouracil sensitivity in esophageal cancer cells. J Exp Clin Cancer Res : CR. 2020;39:131. 32653028 10.1186/s13046-020-01610-1 PMC7353690

[61] Lin K, Jiang H, Zhuang SS, Qin YS, Qiu GD, She YQ, et al. Long noncoding RNA LINC00261 induces chemosensitization to 5-fluorouracil by mediating methylation-dependent repression of DPYD in human esophageal cancer. FASEB J : Publ Federation Am Societies Exp Biol. 2019;33:1972–88. 10.1096/fj.201800759R 30226808

[62] Yang R, Wan J, Ma L, Zhou F, Yang Z, Li Z, et al. TMEM44-AS1 promotes esophageal squamous cell carcinoma progression by regulating the IGF2BP2-GPX4 axis in modulating ferroptosis. Cell Death Discov. 2023;9:431. 38040698 10.1038/s41420-023-01727-0 PMC10692126

[63] Pan C, Chen G, Zhao X, Xu X, Liu J. lncRNA BBOX1-AS1 silencing inhibits esophageal squamous cell cancer progression by promoting ferroptosis via miR-513a-3p/SLC7A11 axis. Eur J Pharmacol. 2022;934:175317. 36216119 10.1016/j.ejphar.2022.175317

[64] Zhang B, Bao W, Zhang S, Chen B, Zhou X, Zhao J, et al. LncRNA HEPFAL accelerates ferroptosis in hepatocellular carcinoma by regulating SLC7A11 ubiquitination. Cell Death Dis. 2022;13:734. 36008384 10.1038/s41419-022-05173-1 PMC9411508

[65] Zhang Y, Luo M, Cui X, O’Connell D, Yang Y. Long noncoding RNA NEAT1 promotes ferroptosis by modulating the miR-362-3p/MIOX axis as a ceRNA. Cell Death Differ. 2022;29:1850–63. 35338333 10.1038/s41418-022-00970-9 PMC9433379

[66] Qi W, Li Z, Xia L, Dai J, Zhang Q, Wu C, et al. LncRNA GABPB1-AS1 and GABPB1 regulate oxidative stress during erastin-induced ferroptosis in HepG2 hepatocellular carcinoma cells. Sci Rep. 2019;9:16185. 31700067 10.1038/s41598-019-52837-8 PMC6838315

[67] He GN, Bao NR, Wang S, Xi M, Zhang TH, Chen FS. Ketamine Induces Ferroptosis of Liver Cancer Cells by Targeting lncRNA PVT1/miR-214-3p/GPX4. Drug Des, Dev Ther. 2021;15:3965–78. 10.2147/DDDT.S332847 PMC8458041 34566408

[68] Huang K, Yu L, Lu D, Zhu Z, Shu M, Ma Z. Long non-coding RNAs in ferroptosis, pyroptosis and necroptosis: from functions to clinical implications in cancer therapy. Front Oncol. 2024;14:1437698. 39267831 10.3389/fonc.2024.1437698 PMC11390357

[69] Gong H, Li Z, Wu Z, Lian G, Su Z. Modulation of ferroptosis by non‑coding RNAs in cancers: Potential biomarkers for cancer diagnose and therapy. Pathol, Res Pract. 2024;253:155042. 38184963 10.1016/j.prp.2023.155042

[70] Fan X, Liu F, Wang X, Wang Y, Chen Y, Shi C, et al. LncFASA promotes cancer ferroptosis via modulating PRDX1 phase separation. Sci China Life Sci. 2024;67:488–503. 37955780 10.1007/s11427-023-2425-2

[71] Mao C, Wang X, Liu Y, Wang M, Yan B, Jiang Y, et al. A G3BP1-Interacting lncRNA Promotes Ferroptosis and Apoptosis in Cancer via Nuclear Sequestration of p53. Cancer Res. 2018;78:3484–96. 29588351 10.1158/0008-5472.CAN-17-3454 PMC8073197

[72] Yao ZY, Xing C, Cai S, Xing XL. Development and Validation of Ferroptosis-Related lncRNAs as Prognosis and Diagnosis Biomarkers for Breast Cancer. BioMed Res Int. 2022;2022:2390764. 36303582 10.1155/2022/2390764 PMC9596248

[73] Qiu X, Shi Q, Zhang X, Shi X, Jiang H, Qin S. LncRNA A2M-AS1 Promotes Ferroptosis in Pancreatic Cancer via Interacting With PCBP3. Mol Cancer Res : MCR. 2022;20:1636–45. 35920801 10.1158/1541-7786.MCR-22-0024

[74] Fu H, Zhang Z, Li D, Lv Q, Chen S, Zhang Z, et al. LncRNA PELATON, a Ferroptosis Suppressor and Prognositic Signature for GBM. Front Oncol. 2022;12:817737. 35574340 10.3389/fonc.2022.817737 PMC9097896

[75] Chen H, Wang L, Liu J, Wan Z, Zhou L, Liao H, et al. LncRNA ITGB2-AS1 promotes cisplatin resistance of non-small cell lung cancer by inhibiting ferroptosis via activating the FOSL2/NAMPT axis. Cancer Biol Ther. 2023;24:2223377. 37370246 10.1080/15384047.2023.2223377 PMC10308868

[76] Tong X, Yu Z, Xing J, Liu H, Zhou S, Huang Y. et al. LncRNA HCP5-encoded protein regulates ferroptosis to promote the progression of triple-negative breast cancer. Cancers. 2023;15:1880. 36980766 10.3390/cancers15061880 PMC10046773

[77] Li H, Wei Y, Wang J, Yao J, Zhang C, Yu C, et al. Long Noncoding RNA LINC00578 Inhibits Ferroptosis in Pancreatic Cancer via Regulating SLC7A11 Ubiquitination. Oxid Med Cell Longev. 2023;2023:1744102. 36846713 10.1155/2023/1744102 PMC9950792

[78] Wang S, Chen J, Li P, Chen Y. LINC01133 can induce acquired ferroptosis resistance by enhancing the FSP1 mRNA stability through forming the LINC01133-FUS-FSP1 complex. Cell Death Dis. 2023;14:767. 38007473 10.1038/s41419-023-06311-z PMC10676390

[79] Wang J, Jia Q, Jiang S, Lu W, Ning H. POU6F1 promotes ferroptosis by increasing lncRNA-CASC2 transcription to regulate SOCS2/SLC7A11 signaling in gastric cancer. Cell Biol Toxicol. 2024;40:3. 38267746 10.1007/s10565-024-09843-y PMC10808632

[80] Yang H, Hu Y, Weng M, Liu X, Wan P, Hu Y, et al. Hypoxia inducible lncRNA-CBSLR modulates ferroptosis through m6A-YTHDF2-dependent modulation of CBS in gastric cancer. J Adv Res. 2022;37:91–106. 35499052 10.1016/j.jare.2021.10.001 PMC9039740

[81] Huang G, Xiang Z, Wu H, He Q, Dou R, Lin Z, et al. The lncRNA BDNF-AS/WDR5/FBXW7 axis mediates ferroptosis in gastric cancer peritoneal metastasis by regulating VDAC3 ubiquitination. Int J Biol Sci. 2022;18:1415–33. 35280682 10.7150/ijbs.69454 PMC8898362

[82] Balihodzic A, Prinz F, Dengler MA, Calin GA, Jost PJ, Pichler M. Non-coding RNAs and ferroptosis: potential implications for cancer therapy. Cell Death Differ. 2022;29:1094–106. 35422492 10.1038/s41418-022-00998-x PMC9177660

[83] Gai C, Liu C, Wu X, Yu M, Zheng J, Zhang W, et al. MT1DP loaded by folate-modified liposomes sensitizes erastin-induced ferroptosis via regulating miR-365a-3p/NRF2 axis in non-small cell lung cancer cells. Cell Death Dis. 2020;11:751. 32929075 10.1038/s41419-020-02939-3 PMC7490417

[84] Ni T, Huang X, Pan S, Lu Z. Inhibition of the long non-coding RNA ZFAS1 attenuates ferroptosis by sponging miR-150-5p and activates CCND2 against diabetic cardiomyopathy. J Cell Mol Med. 2021;25:9995–10007. 34609043 10.1111/jcmm.16890 PMC8572773

[85] Spanos M, Gokulnath P, Chatterjee E, Li G, Varrias D, Das S. Expanding the horizon of EV-RNAs: LncRNAs in EVs as biomarkers for disease pathways. Extracell. Vesicle. 2023;2:100025. 38188000 10.1016/j.vesic.2023.100025 PMC10768935

[86] Huang P, Wang L, Li Q, Tian X, Xu J, Xu J, et al. Atorvastatin enhances the therapeutic efficacy of mesenchymal stem cells-derived exosomes in acute myocardial infarction via up-regulating long non-coding RNA H19. Cardiovascular Res. 2020;116:353–67. 10.1093/cvr/cvz139 PMC8204482 31119268

[87] Chen H, Wang L, Zeng X, Schwarz H, Nanda HS, Peng X, et al. Exosomes, a New Star for Targeted Delivery. Front Cell Developmental Biol. 2021;9:751079. 10.3389/fcell.2021.751079 PMC8531489 34692704

